# Genetically Modified Sugarcane Intercropping Soybean Impact on Rhizosphere Bacterial Communities and Co-occurrence Patterns

**DOI:** 10.3389/fmicb.2021.742341

**Published:** 2021-12-09

**Authors:** Beilei Wei, Jinlian Zhang, Rushuang Wen, Tingsu Chen, Ningshao Xia, Yue Liu, Ziting Wang

**Affiliations:** ^1^State Key Laboratory of Molecular Vaccinology and Molecular Diagnostics, National Institute of Diagnostics and Vaccine Development in Infectious Diseases, School of Life Sciences, School of Public Health, Xiamen University, Xiamen, China; ^2^College of Agronomy, Guangxi University, Nanning, China; ^3^Guangxi Key Laboratory of Sugarcane Biology, Nanning, China; ^4^Microbiology Research Institute, Guangxi Academy of Agricultural Sciences, Nanning, China

**Keywords:** intercropping, transgenic crops, rhizosphere microbial environment, interaction, sugarcane

## Abstract

Strategies involving genes in the dehydration-responsive element binding (DREB) family, which participates in drought stress regulation, and intercropping with legumes are becoming prominent options in promoting sustainable sugarcane cultivation. An increasing number of studies focusing on root interactions in intercropping systems, particularly involving transgenic crops, are being conducted to better understand and thus, harness beneficial soil microbes to enhance plant growth. We designed experiments to investigate the characteristics of two intercropping patterns, soybean with wild-type (WT) sugarcane and soybean with genetically modified (GM) Ea-DREB2B-overexpressing sugarcane, to assess the response of the rhizosphere microbiota to the different cropping patterns. Bacterial diversity in the rhizosphere microbial community differed between the two intercropping pattens. In addition, the biomass of GM sugarcane that intercropped with soybean was significantly improved compared with WT sugarcane, and the aboveground biomass and root biomass of GM soybean intercropping sugarcane increased by 49.15 and 46.03% compared with monoculture. Furthermore, a beneficial rhizosphere environment for the growth of *Actinobacteria* was established in the systems intercropped with GM sugarcane. Improving the production mode of crops by genetic modification is a key strategy to improving crop yields and provides new opportunities to further investigate the effects of intercropping on plant roots and soil microbiota. Thus, this study provides a basis for selecting suitable sugarcane–soybean intercropping patterns and a theoretical foundation for a sustainable sugarcane production.

## Introduction

Sugarcane (*Saccharum officinarum* L.) is a tall perennial grass that stores high concentrations of sucrose in its stems. It is cultivated in over 80 countries in the tropics, semi-subtropics, and subtropics (Tew and Cobill, 2008). As a C4 plant, sugarcane yields a greater biomass than maize, silvergrass (*Miscanthus*), and switchgrass (*Panicum virgatum*) (Heaton et al., 2008). Owing to this high biomass yield, sugarcane cultivation competes less for land designated for food crops and it is the first choice for high-yielding sugar crops (Peskett et al., 2007). However, irregular perennial precipitation and incomplete irrigation facilities in sugarcane regions have made drought one of the main factors restricting sugarcane yield increase; thus, breeding drought-resistant sugarcane varieties has become an urgent task (Santos et al., 2019). Drought resistance itself is a complex trait controlled by many genes (Budak et al., 2015), and their identification will be of great significance for the development and subsequent cultivation of drought-resistant sugarcane varieties (Zhao et al., 2020c). Members of the dehydration-responsive element binding (*DREB*) subfamily of genes play key roles in plant stress responses to low temperatures, drought, and high salinity (Zhou Y. et al., 2019). *Ea-DREB2B*, a member of the *DREB2* family that was cloned from the hardy sugarcane *Saccharum arundinaceum*, plays a critical role in enhancing the tolerance of plants to drought and salinity (Ali et al., 2017). *Ea-DREB2B*-modified transgenic crops have been demonstrated to have improved drought tolerance by altering their plant hormone metabolism and root growth (Paul and Roychoudhury, 2018). It was also speculated that the expression of *Ea-DREB2B* in the roots of transgenic sugarcane affects the production of root exudates and consequently, alters the bacterial community structure in crop rhizosphere (Zhao et al., 2020a).

Intercropping is a practice of planting two or more different crop varieties at the same time in different combinations in the same area of land to improve crop yields; it is commonly implemented for co-cultivation of soybean and gramineous crops (Singh et al., 2018). Intercropping improves the mobilization and absorption of potassium, phosphorus, and micronutrients through rhizosphere interactions, improved soil microecology, and increased microbial counts and enzyme activity in the soil, which are critical to increase crop yield (Zhou Q. et al., 2019). Wang et al. (2020) indicated that soybean and sugarcane intercropping, complemented with reduced nitrogen application could improve sugarcane yield and reduce carbon footprint in China. This practice has been widely used to reduce nitrogen leaching and stabilize yield (Luo et al., 2016; Lian et al., 2018). In addition, previous studies have demonstrated that soybean has a higher impact on biological and chemical properties of soil in intercropping systems compared with other legumes, such as peanuts, thus, ameliorating field ecological conditions and enhancing soil fertility, which favor sugarcane growth (Solanki et al., 2017; Du et al., 2018). However, the impact of intercropping soybean with *DREB* gene-modified crops particularly with drought-tolerant sugarcane varieties on nitrogen uptake remains unclear.

Rhizosphere microbes are essential components of the plant and soil environment and provide valuable ecosystem services. These microbes are associated with numerous important biochemical reactions that affect plant growth and metabolism (Souza et al., 2015). The integration of transgenic plants may have either intended or unintended effects on soil microbial communities and functions (DeBruyn et al., 2017; Wei et al., 2020). The interaction between genetically modified (GM) crops and rhizosphere microbes is mainly associated with the production of root exudates and tiller or leaf degradation (Khan et al., 2017). Accordingly, alterations in the enzymatic activities of rhizosphere microbes have been identified (Chen et al., 2018). Previous studies have indicated that genetic modification can change the rhizosphere bacterial community of plants. Compared with the wild-type (WT) sugarcane, the diversity and composition of bacterial rhizosphere community of *Ea-DREB2B* transgenic sugarcane is significantly different, which has been contributed to the root exudates of the transgenic plants (Zhao et al., 2020a). However, to date, no studies have determined whether the intercropping of transgenic crops with soybean modifies the microbiome composition of the rhizoplane and rhizosphere of soybean.

Metagenomic analysis is used to construct metagenomic libraries by directly extracting all microbial DNA from soil samples and using genomics research strategies to study the genetic composition and community functions of all microorganisms contained in environmental samples (Riesenfeld et al., 2004). Amplicon sequencing and whole genome sequencing are the most widely used in metagenomic analysis (Petrosino et al., 2009). Amplicon sequencing was used to analyze the composition and abundance of microbial communities in environmental samples by using PCR to amplify marker genes shared by microorganisms (Schöler et al., 2017). Commonly used studies include the composition of bacterial archaea based on 16S rRNA gene sequencing, the composition of eukaryotes such as fungi and protozoa studied by ITS/18S sequencing, and the nitrogen fixation related microbial communities studied by nifH gene sequencing (Izquierdo and Nüsslein, 2006; Li et al., 2011; Zhou et al., 2017). The advantages of amplicon sequencing are simple, rapid, low cost and mature analytical methods, which are favored by many microbial researchers. Illumina Hiseq2,500 and MiSeq are commonly used sequencing platforms (El-Metwally et al., 2014). At present, the main analysis processes of ampland data analysis have been quite mature, including data preprocessing (combination or splicing of double-ended sequences, data quality control, chimera removal), OTU sequence clustering, α diversity analysis, β diversity analysis, etc. With the continuous development of sequencing technology, more and more microbial sequence data will be generated. How to efficiently and quickly analyze data becomes particularly important. Under this demand, amplicon analysis platforms emerged at the historic moment. Currently, the three mainstream analysis platforms are Mothur (Schloss et al., 2009), QIIME (Caporaso et al., 2010), and USEARCH (Edgar, 2010). Mothur is a bioinformatics software with a very good architecture, integrating a large number of tools and modules, and standardizing the input and output. It can be used for distance calculation and diversity calculation, which is very suitable for the study of microbial ecology and population structure (Beck et al., 2015). USEARCH integrates many classical sequence analysis algorithms, most of which start with U, such as UCLUST sequence clustering algorithm, UPARSE algorithm, UNOISE sequence quality control algorithm and UCHIME removal algorithm. At present, USEARCH software has related commands in sequence quality control, chimeric removal, sequence search, OTU clustering and other processes, which are widely used (Prodan et al., 2020).

In this study, we conducted polymerase chain reaction (PCR) and high-throughput sequencing analyses to characterize the rhizosphere microbial communities in soybean fields intercropped with WT and GM sugarcane. We hypothesized that intercropping of GM sugarcane and soybean would affect the rhizosphere ecology and promote crop growth through changes in the rhizosphere microbial community structure instigated by GM sugarcane. Specifically, we aimed to: (1) determine whether the *DREB*-modified sugarcane and the WT sugarcane have different effects when intercropped with soybean, (2) establish the effect of GM sugarcane and soybean intercropping on the rhizosphere environment, and (3) characterize the relationship between the changes in the rhizosphere bacterial community and root growth. We believe that our study will provide a new idea for the design of planting pattern of sugarcane, that is, when interplanting with soybean, the choice of GM sugarcane interplanting is of positive significance to the formation of good soil environment.

## Materials and Methods

### Plants and Field Experimental Design

This study was conducted in the forage breeding ground of Guangxi University located in Fusui, China (22°17′0.01″N, 108°06′0.01″E). The experimental field was established on a sugarcane continuous cropping field. The soil was of lateritic red earth, with total nitrogen 0.093%, total phosphorus 0.024%, total potassium 0.500%, available nitrogen 70 mg kg^–1^, available phosphorus 10 mg kg^–1^, available potassium 286 mg kg^–1^, organic matter 8.90 g kg^–1^, and pH 8.33 in the topsoil.

GN18 modified with a DREB gene (GM sugarcane) and FN95–1702 (WT sugarcane) were used as the two sugarcane cultivars in this experiment, and a local widely planted soybean cultivar, GUIZAO2, was used. The GN18 sugarcane cultivar was generated from FN95–1702 by transforming the inducible promoter rd29A into sugarcane callus via particle bombardment to overexpress *Ea-DREB2B* (Huaizhu et al., 2004). Five planting modes were designed: monocropped FN95–1702 (WT) sugarcane, monocropped GN18 (GM) sugarcane, monocropped GUIZAO2 soybean, FN95–1702 sugarcane intercropped with GUIZAO2 soybean, and GN18 sugarcane intercropped with GUIZAO2 soybean. All experiments were conducted in triplicates in each plot.

**FIGURE 1:**
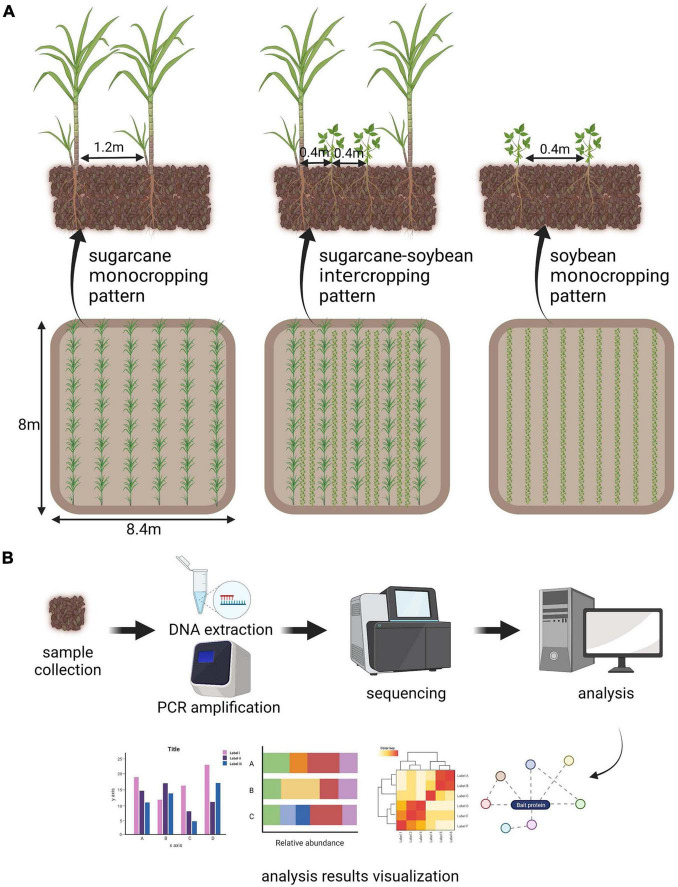
**(A)** Self-interpretation diagram of experimental design. **(B)** Simple explanation of metagenomic analysis process.

In the sugarcane–soybean intercropping and sugarcane monocropping systems, eight sugarcane rows, 8 m in length were planted at 1.2 m spacing and two rows of soybean were planted between sugarcane rows, with 0.4 m row spacing and 0.2 m plant spacing. Soybean monocropping plot consisted of 20 rows with a length of 8 m and row spacing of 0.4 m. The five cropping systems were established on single plots, each with an area of 67.2 m^2^. We made Figure 1A to understand our experimental setup. Sugarcane was planted in March 2018, and soybean was planted 15 d after sugarcane plantation. Sugarcane stem segments and soybean seeds were soaked in 3% H_2_O_2_ to disinfect their surfaces, and then rinsed thrice with deionized water before planting and sowing (Mahakham et al., 2017). After 45 days, whole sugarcane and soybean plants were collected along with the rhizosphere.

### Crop Sample Collection and Determination of Biomass

Crop samples were randomly collected from the sugarcane mono-cropping plot (hereafter referred to as Mono–Sug), soybean monocropping plot (Mono–Soy), and sugarcane-soybean intercropping plot (Inter–Sug and Inter–Soy), sugarcane samples in soybean sugarcane intercropping are called Inter-Sug, including plant samples and rhizosphere soil samples; similarly, soybean samples in soybean and sugarcane intercropping are called Inter-Soy. Six plants were sampled per experimental plot of each treatment. The aboveground and underground parts of sugarcane and soybean were cleaned and their dry weight was determined by drying in the oven (Cui et al., 2018).

### Soil Sample Collection and Physicochemical Analysis

The samples of soil were collected from the sugarcane rhizosphere in the Mono–Sug plots, soybean rhizosphere in the Mono–Soy plots, and sugarcane rhizosphere and soybean roots in the Inter–Sug and Inter–Soy plots, respectively. The roots were gently shaken to disengage the loosely attached soil on the root, and the soil thereafter attached to the root surface was defined as rhizosphere soil. This rhizosphere soil was removed by brushing with a sterilized brush and collected in a sterile bag. Collected rhizosphere soil sample from the same place was used as a composite sample. A 2 mm sieve was used to remove impurities, including plant residues and stones, from the soil samples and the resultant samples were divided into two portions, one for environmental factor determination and the other for DNA extraction. Later, the samples were stored at -80 °C until further use. Available phosphorus (AP) and soil organic carbon (SOC) were determined following the methodologies mentioned by Jackson (1969) and Soon and Abboud (1991), respectively, and total nitrogen (TN) was determined using a semimicro Kjeldahl method (Bremner and Tabatabai, 1972).

### DNA Extraction, Amplicon Generation, and High-Throughput Sequencing

DNA was extracted from 1 g soil samples using a DNA extraction kit (Omega Bio-Tek, Inc., Norcross, GA, United States). NanoDrop One spectrophotometer (Thermo Fisher Scientific, Waltham, MA, United States) was used to measure the DNA concentration and purity. A partial sequence of the 16S rRNA gene was amplified using a specific primer pair (338F–806R, F: ACTCCTACGGGAGGCAGCAG; R: GGACTACHVGGGTWTCTAAT) (Sun et al., 2020) that contained a 12 bp barcode; the primer pair was synthesized by Sangon (Sangon biotech, Shanghai, China). PCR reaction mixtures, containing 25 μL of 2 × Premix Taq (Sangon biotech, Shanghai, China), 2 μL of each primer (0.4 μM), and 1 μL of DNA template (20 ng μL^–1^) in a total volume of 50 μL, were amplified by thermocycling under the following conditions: 4 min at 94°C for initial denaturation, followed by 35 cycles of 30 s denaturation at 94°C, 30 s annealing at 60°C, and extension at 72°C for 30 s, and final extension at 72°C for 10 min. Amplification was carried out in a thermocycler (Biometra, Goettingen, Germany). Illumina TruSeq DNA sample preparation kit (Illumina, San Diego, CA, United States) was used for the construction of DNA libraries. Illumina HiSeq 2,500 platform (Illumina, San Diego, CA, United States) was used to perform high-throughput sequencing of amplified 16S rRNA sequences, and 250 bp of paired-end reads were obtained. There is a simple flow chart to understand our metagenomic analysis process (Figure 1B).

### Bioinformatics

Mothur (version 1.35.1) was used to assign the original tag sequence to unique barcodes and primers to obtain clean reads (Schloss et al., 2009). Further, to acquire tags, FLASH (version 1.2.7)^1^ was used to pair clean reads (Liang et al., 2019) and USEARCH (Version 8.1.1861)^2^ was used to analyze the sequence. The 16S rRNA gene sequence was clipped to a fixed length of 360 bp, sorted by abundance, de-replicated, and clustered using UPARSE (version 7.1)^3^ to determine operational taxonomic units (OTUs) with a 97% similarity (Chen et al., 2020). UCHIME (v4.2.4,025)^4^ was used to remove chimeric sequences against the GOLD database (Sekoai et al., 2020). SILVA database was used for taxonomies classification (Bai et al., 2020).

### Diversity Analysis

Alpha diversity was investigated using QIIME (version 1.9.1) (Caporaso et al., 2010). R software (version 2.15.3) was used to display the diversity of samples (R Core Team, 2011). Shannon index and Chao1 index were used to identify community diversity. The data involved are the data fitting of 6 replicate samples to ensure the reliability of the results. Beta diversity was analyzed using principal coordinate analysis (PCoA) based on the Bray-Curtis distance to show the differences between the bacterial community species composition (Tai et al., 2015). The filtered OTU sequence count was normalized using the “trimmed means of M” (TMM), and the normalized results were counted as “count per million” (CPM) (Lee et al., 2016; Fernández-González et al., 2020). We used permutational multivariate analysis of variance (PERMNOVA) to investigate the effects of sample types and intercropping patterns on community differences. Analysis of similarity (ANOSIM) was used to determine the significance of differences in the bacterial community structure under different planting patterns. The correlation between the host root biomass and the rhizosphere compositional similarity was determined using the Mantel test implemented in the Vegan package in R (Hernández et al., 2020).

### Bipartite and Co-occurrence Networks

Indicator analysis for significant planting patterns (*p* < 0.05) was conducted using bipartite network visualization. For all networks, we used the TMM normalization, and Spearman’s correlation analysis between the OTUs. In addition, we used Spearman’s correlation analysis to assess the association between environmental factors and rhizosphere bacteria to determine the topological network attributes. For this, we included TMM-standardized CPM bacterial count in the OTU table of soil and root communities and used Spearman’s rank correlation analysis to evaluate the correlation between the bacterial OTUs. The network properties were summarized and analyzed, network modules were identified, and the community structure of each plant unit network was analyzed (Hartman et al., 2018). OTUs with network node degree values in the top 1% of nodes in each planting mode were identified as the key meta-network OTUs. We prioritized this simple definition over the other complex definition because both definitions essentially reveal the same keystone OTUs. Further, the correlation between the modules and soil environmental factors was investigated through the Mantel test (Tai et al., 2020).

## Results

### Comparison of Biomass and Soil Nutrients Between Two Intercropping Models

Compared with those of monocropped soybean (Table 1), both WT and GM sugarcane intercropping patterns increased the aboveground and root biomass of soybeans significantly; the GM sugarcane-soybean intercropping pattern produced the highest soybean aboveground and root biomass. Compared with WT, GM sugarcane had a higher aboveground biomass and root biomass when intercropped with soybean. In terms of soil nutrients, the SOC, TN, and AP contents were higher in Inter–Soy than in Mono–Soy. The SOC and TN contents were higher, but the AP content was lower in GM intercropping than in WT intercropping systems.

**TABLE 1:** Soil characteristics of different patterns.

Sample	Root biomass (g)	Above ground biomass(g)	SOC (g⋅kg^–1^)	TN (g⋅kg^–1^)	AP (g⋅kg^–1^)
Mono-soy	7.15c	29.10d	5.32d	0.537a	3.51c
WT Mono-sug	24.24b	118.44b	5.23d	0.369a	4.67ab
WT Inter-sug	31.20ab	147.60ab	7.18bc	0.400a	5.83a
WT Inter-soy	8.50c	34.95d	5.59cd	0.393a	5.63a
GM Mono-sug	25.04b	82.56bc	5.73cd	0.505a	2.39d
GM Inter-sug	35.40a	176.16a	9.84a	0.707a	4.03bc
GM Inter-soy	10.85c	44.35cd	7.67b	0.622a	3.85bc

*Different letters indicate significant differences (ANOVA, P < 0.05).*

### Rhizosphere Community Diversity Under Genetically Modified and Wild-Type Sugarcane Intercropping With Soybean

**FIGURE 2:**
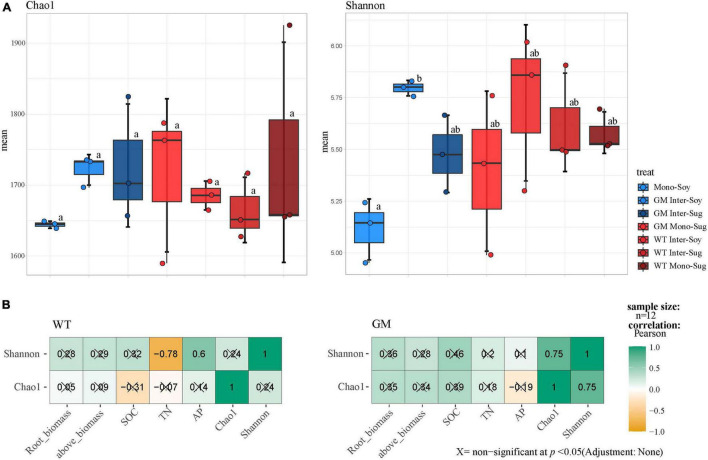
**(A)** The results of Chao 1 index and Shannon index showed the α-diversity of bacteria. F, Fisher’s F-ratio. *p*, *p*-value. The letters next to the bar chart indicate the significant differences between the processed data. **(B)** Pearson analysis of the correlation between bacterial α-diversity and environmental factors.

Alpha diversity analysis revealed that intercropping improved the rhizosphere microbial diversity and abundance of soybean compared with monocropping, and the differences in the Chao1 index between WT Inter–Soy and WT Inter–Sug were not significant. Significant difference in the Shannon index was registered only between GM sugarcane–soybean intercropping and soybean monocropping (Figure 2A). The Pearson’s correlation analysis of the alpha diversity and nutrients showed that in the WT sugarcane–soybean intercropping pattern, the Shannon index was negatively correlated with TN and positively correlated with AP, whereas the Shannon and Chao1 indexes were significantly correlated with each other, but not with the soil environmental factors (Figure 2B).

**FIGURE 3:**
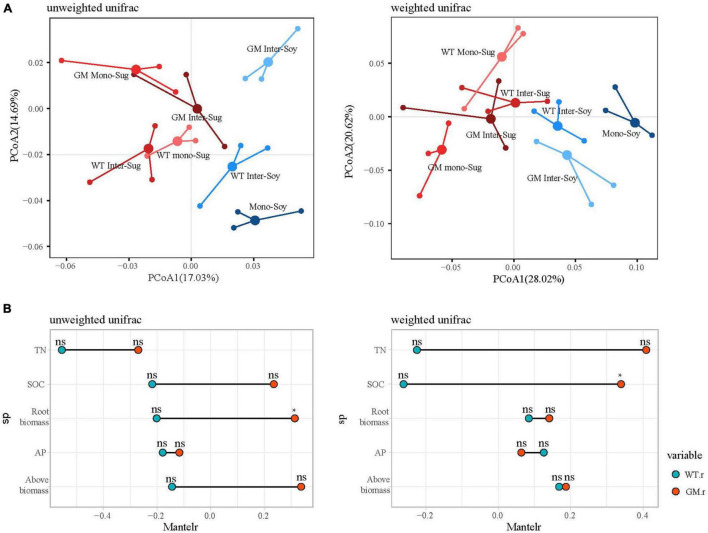
**(A)** Principal coordinate analysis (PCOAS) based on the Unifrac distance matrix indicated differences in bacterial beta diversity under different planting modes. **(B)** Mantel test compared the relative differences of soil nutrients under different planting modes. *P* > 0.05(NS); *P* < 0.05(*).

Beta diversity analysis for the two intercropping patterns was presented by PCoA of unweighted and weighted UniFrac distance matrices (Figure 3A). PCoA of the unweighted and weighted UniFrac distances revealed a significant difference between the GM sugarcane–soybean and the WT sugarcane–soybean intercropping patterns. The unweighted-based PCoA showed a larger significant difference in the soybean rhizosphere between GM sugarcane–soybean intercropping and monocropped soybean; after weighting, no significant difference in the soybean rhizosphere bacteria was observed between the two intercropping patterns. According to the Mantel test under the unweighted conditions, the two intercropping patterns showed significant differences in TN, SOC, root biomass, and aboveground biomass, with GM being more strongly correlated with TN, SOC, and biomass (Figure 3B). After weighting, the two intercropping patterns showed significant differences in TN and SOC, and the GM sugarcane was more significantly correlated with TN and SOC.

**FIGURE 4:**
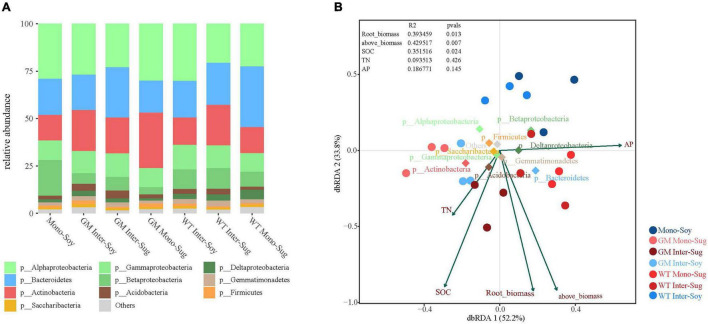
**(A)** Relative abundance of different phyla in each pattern. **(B)** Distance-based Redundancy analysis of different patterns (dot), abundant classes (rhombus), and environmental factors (arrows) indicates the dominant communities and influential environmental factors.

Compared with the monocropped soybean plots, there was a higher relative abundance of Actinobacteria and a lower relative abundance of Betaproteobacteria in the GM intercropping pattern. The comparison of soybean intercropping patterns revealed a higher relative abundance of Actinobacteria in the GM sugarcane–soybean intercropping pattern, and a higher relative abundance of Betaproteobacteria in soybean intercropping with WT sugarcane (Figure 4A). In addition, compared with WT Mono-Sug, GM sugarcane intercropping increased the relative abundance of Actinobacteria, but reduced the relative abundance of Bacteroidetes. GM Inter–Soy had a higher relative abundance of Actinobacteria, but a lower relative abundance of Betaproteobacteria compared with Mono–Soy. The comparison of the two intercropping patterns revealed a higher relative abundance of Actinobacteria in GM Inter–Soy, and a higher relative abundance of Betaproteobacteria in WT Inter–Soy (Figure 4A). In addition, GM Mono–Sug also had a higher relative abundance of Actinobacteria, but a lower relative abundance of Bacteroidetes compared with that of WT Mono–Sug. The correlation between environmental factors and each component (Mono–Soy, Inter–Soy, Inter–Sug, and Mono–Sug) in the two intercropping patterns was further explored through dbRDA analysis. The first two axes of dbRDA accounted for 52.2 and 33.8% of the total variation, respectively, for the two intercropping patterns (Figure 4B). AP was highly correlated with spindle 1, while SOC was highly correlated with spindle 2. Compared with GM Inter–Soy, Mono–Soy showed a large difference in distance, and was mainly influenced by TN and SOC. The distributions of WT and GM components were significantly different.

### Genetically Modified and Wild-Type Sugarcane Intercropping Patterns Produced Different Rhizosphere Co-occurrence Network Interactions

**FIGURE 5:**
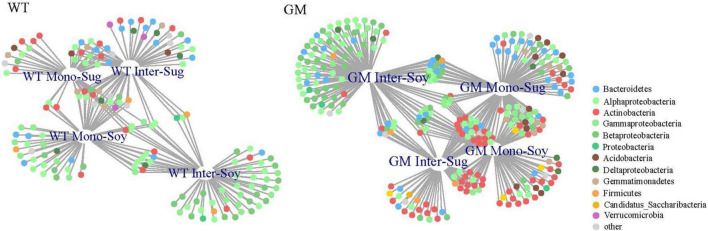
Bipartite networks in the rhizosphere bacterial community shows the specific planting system OTUs. The circles represented single bacterial OTU, which were positively and significantly associated with one or more cropping methods (*P* < 0.05). Different colors represent different phyla to which they belong.

**FIGURE 6:**
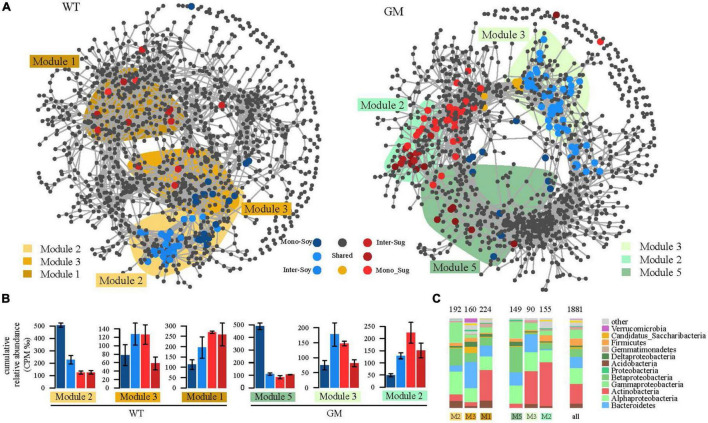
**(A)** Microbial symbiosis of transgenic and wild-type sugarcane root system under different Yuan intercropping combinations. The co-occurrence network visualized the correlation between operational taxa (OTUs) (ρ > 0.7, *p* < 0.001). These colored dots represent OTUs belonging to different planting patterns. Gray dots indicate insensitivity to experimental treatment. The shaded area represents the network module that contains the OTU for each response. **(B)** Cumulative relative abundance (as counts per million, CPM; y-axis in × 1,000) of all bacteria of the cropping sensitive modules in networks. The cumulative relative abundance in samples of Inter_Soybean (light blue), Mono_Soybean (dark blue), Inter_Sugercane (dark red), Mono_Sugarcane (light red) cropping systems indicates the overall response of cropping sensitive modules to the different patterns. **(C)** The composition of the relative abundance at the class level in each module is compared with the overall taxonomic distribution of the entire dataset (the “ALL” column).

**FIGURE 7:**
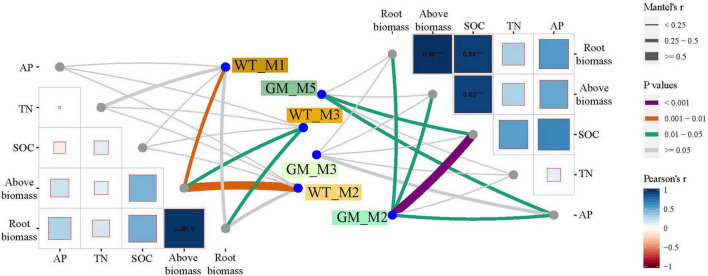
Mantel test is the correlation analysis between modules obtained by network and edaphic physicochemical factors. The color of lines reflects the significance between data groups, and the thickness of lines reflects the correlation between data groups.

A bipartite network was used to analyze the cluster relation of the responsive OTUs in the two intercropping patterns. More responsive OTUs were clustered, and the components were more closely related to each other in the GM intercropping pattern than in the WT intercropping pattern. The GM sugarcane–soybean intercropping pattern could cluster more Actinobacteria (Figure 5). By analyzing OTUs of the corresponding treatments in the two intercropping modes at the genus level, it was found that there were more OTUs responding to GM sugarcane and soybean intercropping pattern, and the corresponding genera were also different. For example, the top three genera responding to GM Inter–Sug. *Taibaiella* (10), *Rhodanobacter* (9) and *Sphingomonas* (7); *Gemmatimonas* (4), *Nocardioides* (4), and *Lysobacter* (3) responded to WT intercropping pattern. In addition, *Sphingomonas* had higher response numbers in both GM Inter–Sug and GM Mono–Sug. *Gemmatimonas* and *Nocardioides* had higher response numbers in both WT Inter–Sug and WT Mono–Sug, and *Gemmatimonas* also had more OTUs responses in WT Inter–Soy (Supplementary Tables 1, 2 and Supplementary Figure 2). The co-occurrence network analysis revealed a significant correlation between the OTUs in the rhizosphere bacterial communities under the two intercropping patterns (Figure 6A). In the WT sugarcane-soybean intercropping pattern, module2 (M2) contained OTUs corresponding to Mono–Soy, while module1 (M1) mainly contained OTUs analogous to Inter–Sug and Mono–Sug (Figure 6B). In the GM sugarcane–soybean intercropping pattern, module3 (M3) contained OTUs that corresponded to Inter–Soy, while module2 (M2) and module5 (M5) mainly contained OTUs that corresponded to Inter–Sug and Mono–Sug. Compared with the WT sugarcane–soybean intercropping pattern, more responsive OTUs were detected in the GM intercropping pattern network, and many OTUs corresponded to both soybean and GM sugarcane (Figure 6A). The analysis of responding OTUs in genus level of each module in network analysis showed that *Streptomyces*, *Nocardioides* and *Sphingomonas* were the most responding bacteria in M2 and M5 modules of GM sugarcane soybean intercropping pattern (Supplementary Tables 3, 4). This also verified the result of the cluster analysis of OTUs in the binary network for each treatment in the two intercropping patterns (Supplementary Tables 1, 2). Classifying the OTUs in each module at the phylum level revealed a large difference between the two intercropping patterns—WT had a higher relative abundance of Bacteroidetes, while GM had a higher relative abundance of Actinobacteria (Figure 6C). In the node degree analysis, a higher node degree was observed for OTUs in response to soybean in the WT intercropping and for OTUs in response to sugarcane in the GM intercropping pattern (Supplementary Figure 1). In addition, the results of the Mantel tests revealed that M2 was generally significantly correlated with the biomass and environmental factors, except TN, and significantly correlated with SOC in the GM intercropping pattern. The WT intercropping pattern showed no significant correlation between each module and the environmental factors (Figure 7).

## Discussion

### Soybean Rhizosphere Bacterial Communities Differ Between Genetically Modified and Wild-Type Intercropping Patterns

The alpha diversity results indicated that sugarcane–soybean intercropping increased the diversity of the bacterial community in soybean roots and intercropping with GM sugarcane significantly improved the bacterial diversity of the soybean rhizosphere (Figure 2A). SOC and TN under the GM sugarcane–soybean intercropping pattern significantly improved compared with those under the WT sugarcane–soybean intercropping pattern (Table 1). Based on these findings, we speculated that the root system of GM sugarcane releases more exudates, such as sugars and organic acids, which provide nutrients and vitamins required for bacterial growth, alter soil properties, and thereby promote the development of different rhizosphere microbial communities (Da Costa et al., 2018; Sasse et al., 2018; Pereira et al., 2019). Intercropping can also change the SOC content in the rhizosphere of crops and affect the community structure of rhizosphere bacteria (Zhou Q. et al., 2019). Pearson’s correlation analysis revealed that the WT and GM sugarcane bacterial communities had different relationships with the environmental factors (Figure 2B), which further demonstrated that the two sugarcane varieties may shape different rhizosphere bacterial structures due to their influences on soil environmental factors (Zhang et al., 2016). Direct contact between the roots of two intercropped plants leads to interactions between their root exudates, which in turn affects the bacterial community structure (Gao et al., 2019; Yu et al., 2019). Therefore, we speculated that root exudation may play a positive role in maintaining or modifying the community structure of rhizosphere soil microbes, the enrichment of which may considerably impact plant growth (Huangfu et al., 2019). The beta diversity analysis showed that the composition of the rhizosphere bacterial community differed between the GM sugarcane–soybean and WT sugarcane–soybean intercropping patterns, and that the community structure of the soybean rhizosphere bacteria was modified by the interaction between GM sugarcane and soybean roots (Figure 3A). Thus, the expression of drought resistant genes could enrich the bacterial community and improve the rhizosphere environment compared with those in the WT intercropping pattern (Sayer et al., 2017). The Mantel test indicated that GM sugarcane was more closely related to the environmental factors than was WT, especially to TN and SOC (Figure 3). This indicated that the changes in the rhizosphere soil environment can affect the diversity of the bacterial community (Zhao et al., 2020a). Previous studies have shown that the overexpression of *Ea-DREB2* in GM sugarcane increases the photosynthetic rate and chlorophyll content of sugarcane (Augustine et al., 2015). Plants may release up to 20% of their photosynthetic products into the rhizosphere soil, which provides the nutritional basis for establishing the plant–microbes interactions (Haichar et al., 2008). Rhizosphere bacteria in the soil can release growth-promoting compounds that promote the establishment of compact soil structure, contribute to the dissolution of organic compounds and release of minerals, and interact with plant roots to support plant growth (Igiehon and Babalola, 2018). In addition, the rhizosphere microbiome can have a considerable effect on plant health by enhancing the abiotic stress tolerance of plants (Zancarini et al., 2013). Therefore, we conclude that the drought-tolerant GM sugarcane cultivar secreted and accumulated large amounts of rhizosphere and soil nutrients, which contributed to the evidently different rhizosphere bacterial community of soybean intercropped with the drought-tolerant sugarcane compared with that of soybean intercropped with WT sugarcane.

### Intercropping of Genetically Modified Sugarcane and Soybean Alters the Rhizosphere Soil Environment

Compared with WT sugarcane, cultivation of GM sugarcane increased the abundance of Actinobacteria when monocropped and increased the relative abundance of Actinobacteria in the soybean rhizosphere when intercropped (Figure 4A). Previous studies have demonstrated that in fields intercropped with GM sugarcane and soybean, the GM sugarcane released larger amounts of nutrients into the soybean rhizosphere, which led to a close interaction between the roots of soybean and those of GM sugarcane, an increase in the number of rhizosphere bacterial species, and the expansion of the GM sugarcane rhizosphere (Gong et al., 2019; Zhou Q. et al., 2019). The introduction of drought-resistance genes has been shown to enhance the secretion of root exudates, which further shapes and influences the rhizosphere microbial community of intercropped soybean (Chun-miao et al., 2015). Moreover, a higher diversity of bacterial species around or in the roots plays an important role in the recycling of soil nutrients, and thus, improves soil fertility (Lisuma et al., 2020). Compared with WT sugarcane intercropping, the abundance of Actinobacteria was higher in Inter–Sug (Figure 4A), thus, contributing to higher nitrogen fixation, ferrite synthesis, phytohormone synthesis, and solubilization of minerals, which enhanced their availability to plants (Barka et al., 2016). Peng et al. (2014) reported that sugarcane and soybean intercropping had a significant effect on the diversity of sugarcane rhizosphere nitrogen-fixing bacteria. GM sugarcane improved the rhizosphere microenvironment of soybean by secreting root exudates, altered the specific flora (such as Actinobacteria and Betaproteobacteria), and promoted the growth of soybean (Sasse et al., 2018; Xu et al., 2018). Actinobacteria dominate the microbiota in plant roots owing to the close relationship between roots and soil bacteria (Liu et al., 2019). They have the potential to promote plant growth and are known to play important roles in nitrogen mineralization and breakdown of organic materials, such as chitin and cellulose (Hamdali et al., 2008; Li et al., 2010). The difference in relative abundance of Betaproteobacteria is mainly reflected in the mono-cropping of GM and WT sugarcane. That is, the two sugarcane cultivars enrich different bacteria in the soil, and Betaproteobacteria also has a positive effect on plant growth, Burkholderia secrete organic acids or acid phosphatases to convert insoluble phosphorus in soil into soluble phosphorus that can be directly absorbed and utilized by plants, thus promoting the utilization of soil phosphorus by plants (Peix et al., 2001; Caballero-Mellado et al., 2007). Similarly, Flavobacterium is a genus in Bacteroidetes with higher relative abundance in WT intercropping system, which has the ability of promoting plant growth, biological control and inducing plant systemic resistance, but its abundance in WT intercropping system is still not high (Jaiswal et al., 2017). The dbRDA analysis between the two intercropping patterns and environmental factors revealed significant differences in the correlation between these factors. These differences were mainly affected by the SOC and AP contents (Figure 4B). Previous studies have suggested that plants drive and shape the structure of rhizosphere bacterial communities by secreting specific compounds in root exudates, and that the species enriched in these communities often play a positive role in plant growth (Li et al., 2019; Cao et al., 2020). In addition, the activation of these rhizosphere nutrients is triggered by the activity of rhizosphere microbes (Zheng et al., 2018). The bipartite network revealed higher abundance in clustered OTUs in GM sugarcane–soybean than in WT sugarcane intercropping systems, which indicates that the rhizosphere bacterial community of GM sugarcane is more closely related to soybean, and that GM sugarcane promotes the soybean biomass (Figure 5). A variety of plant growth-promoting rhizobacteria (PGPR) are enriched, thus, changing the rhizosphere environment and activating more nutrients in the soil (Wang et al., 2017; Gouda et al., 2018).

### Intercropping Between Genetically Modified Sugarcane and Soybean Promotes the Growth of These Crops

Differences between GM sugarcane-soybean intercropping and WT sugarcane-soybean intercropping in rhizosphere bacterial community networks revealed that the expression of *DREB* gene enhanced the intraspecific competition between sugarcane cultivars, and made OTUs response of rhizosphere community stronger (Figures 6A,B). This may be related to the fact that the root interaction between soybean and GM sugarcane is stronger than that between soybean and WT sugarcane (Zhao et al., 2020b). This was also confirmed by the node degree analysis (Supplementary Figure 1). Owing to the interaction between sugarcane and soybean roots and their exudates, the bacteria enriched in the transgenic sugarcane intercropping pattern were more closely related than those enriched in the wild-type sugarcane intercropping pattern (Fan et al., 2017). This was further confirmed by the network analysis of GM and WT sugarcane intercropping patterns, which showed a closer connection between the Inter–Sug and Inter–Soy in GM intercropping pattern (Figure 5). Therefore, the bacteria showed a higher population correlation, in other words, the rhizosphere between GM sugarcane and soybean root had higher contact levels (Lareen et al., 2016). In addition, genus such as *Taibaiella* and *Rhodanobacter* that respond to GM sugarcane soybean intercropping have been identified as PGPR and play an important role in hydrocarbon degradation (Zhang et al., 2013; Gutierrez, 2019; Diallo et al., 2021). The GM sugarcane–soybean intercropping pattern significantly increased the biomass these of the two crops compared with that of WT intercropping pattern (Table 1); thus, intercropping of GM sugarcane and soybean will promote the growth of both species. We speculated that the existence of a close interaction between the GM sugarcane and soybean root systems increased the similarity between the soybean and GM sugarcane rhizosphere bacterial species, provided a higher abundance of soil nutrients and promoted soybean root growth. The GM sugarcane rhizosphere harbored a higher abundance of Actinobacteria (Figure 6C). In the soil, Actinobacteria play important roles in the decomposition of refractory biomaterials and the formation of humus (Anandan et al., 2016). Actinobacteria have a variety of characteristics that promote plant growth directly via the production of plant growth hormones (indole-3-acetic acid, cytokinin, gibberellin, and abscisic acid), biological nitrogen fixation, and solubilization (Yadav et al., 2018; Sekaran et al., 2019). The diversity of the GM sugarcane rhizosphere community was significantly correlated with root biomass, and the structural composition of the rhizosphere bacterial community of soybean intercropped with sugarcane was closely correlated with soybean biomass (Figure 7). Many PGPR affect the plant hormone balance and stress responses by altering plant hormone levels (Tsukanova et al., 2017). The enrichment of a variety of PGPR can both alter the microbial community in the soil around soybean root systems and colonize the root systems themselves (Trabelsi and Mhamdi, 2013). The PGPR can synthesize important growth hormones (e.g., auxins, cytokinins, and gibberellins) that directly promote root growth (Lugtenberg et al., 2013), produce iron carriers, or promote nitrogen fixation and phosphate solubilization to improve soil fertility, and indirectly promote root growth (Bhardwaj et al., 2014). Our findings indicated that intercropping between GM sugarcane and soybean can beneficially modify the bacterial community in the soybean rhizosphere, enhance the abundance and diversity of PGPR, improve soil fertility, regulate plant hormone levels in soybean roots, and promote soybean root growth.

## Conclusion

In this study, we investigated the effects of intercropping soybean with GM and WT sugarcane on the rhizosphere bacterial community and biomass of the crops. Intercropping with GM and WT sugarcane resulted in the formation of different rhizosphere bacterial communities, with the former enhancing the number of representative species of Actinobacteria around the roots of soybean. This result is attributed to the production of larger amounts of root exudates by GM sugarcane roots that have a stimulatory effect on rhizosphere bacterial community development. Furthermore, the GM sugarcane intercropping pattern showed a higher microbial population correlation, and had a stronger positive effect on crop growth than the WT sugarcane intercropping pattern. We also found that the roots of soybean interacted closely with those of GM sugarcane, thereby increasing the similarity between the rhizosphere bacterial species compositions of these two crops. Intercropping with GM sugarcane was shown to improve the microbial communities of the rhizosphere, thereby promoting crops root growth. Therefore, the intercropping pattern of GM sugarcane and soybean is of great value for increasing crop yield and improving the microbial environment of rhizosphere soil.

## Data Availability Statement

The datasets presented in this study can be found in online repositories. The names of the repository/repositories and accession number(s) can be found below: https://www.ncbi.nlm.nih.gov/, PRJNA597165.

## Author Contributions

BW: visualization and data curation. JZ: investigation, validation, and formal analysis. RW: writing—review and editing and software. NX and TC: revision. YL: writing—original draft preparation and investigation. ZW: conceptualization and methodology. All authors contributed to the article and approved the submitted version.

## Conflict of Interest

The authors declare that the research was conducted in the absence of any commercial or financial relationships that could be construed as a potential conflict of interest.

## Publisher’s Note

All claims expressed in this article are solely those of the authors and do not necessarily represent those of their affiliated organizations, or those of the publisher, the editors and the reviewers. Any product that may be evaluated in this article, or claim that may be made by its manufacturer, is not guaranteed or endorsed by the publisher.

## Footnotes

1
http://ccb.jhu.edu/software/FLASH/

2
http://www.drive5.com/usearch/

3
http://drive5.com/uparse/

4
http://drive5.com/uchime
