# Corrigendum: Genetically Modified Sugarcane Intercropping Soybean Impact on Rhizosphere Bacterial Communities and Co-occurrence Patterns

**DOI:** 10.3389/fmicb.2021.835633

**Published:** 2022-02-09

**Authors:** Beilei Wei, Jinlian Zhang, Rushuang Wen, Tingsu Chen, Ningshao Xia, Yue Liu, Ziting Wang

**Affiliations:** ^1^State Key Laboratory of Molecular Vaccinology and Molecular Diagnostics, National Institute of Diagnostics and Vaccine Development in Infectious Diseases, School of Life Sciences, School of Public Health, Xiamen University, Xiamen, China; ^2^College of Agronomy, Guangxi University, Nanning, China; ^3^Guangxi Key Laboratory of Sugarcane Biology, Nanning, China; ^4^Microbiology Research Institute, Guangxi Academy of Agricultural Sciences, Nanning, China

**Keywords:** intercropping, transgenic crops, rhizosphere microbial environment, interaction, sugarcane

In the published article, there was an error. The author order was incorrectly listed as “Jinlian Zhang, Beilei Wei, Rushuang Wen, Yue Liu, and Ziting Wang.” The correct order is Beilei Wei, Jinlian Zhang, Rushuang Wen, Tingsu Chen, Ningshao Xia, Yue Liu, and Ziting Wang, In addition, Beilei Wei and Jinlian Zhang are co-first authors. Tingsu Chen and Ningshao Xia were not included as authors in the published article. The author details have been updated and the corrected Author Contributions Statement appears below:

“BW: visualization and data curation. JZ: investigation, validation, and formal analysis. RW: writing—review and editing and software. NX and TC: revision. YL: writing—original draft preparation and investigation. ZW: conceptualization and methodology. All authors contributed to the article and approved the submitted version.”

The authors apologize for this error and state that this does not change the scientific conclusions of the article in any way. The original article has been updated.

## Publisher's Note

All claims expressed in this article are solely those of the authors and do not necessarily represent those of their affiliated organizations, or those of the publisher, the editors and the reviewers. Any product that may be evaluated in this article, or claim that may be made by its manufacturer, is not guaranteed or endorsed by the publisher.

