# Supplementary material for: Genetically Modified Sugarcane Intercropping Soybean Impact on Rhizosphere Bacterial Communities and Co-occurrence Patterns
Source: Front Microbiol. 2021 Dec 9;12:742341. doi: 10.3389/fmicb.2021.742341 (PMC8713472; doi:10.3389/fmicb.2021.742341)
Supplement: Supplementary file 5 [file Data_Sheet_6.PDF]

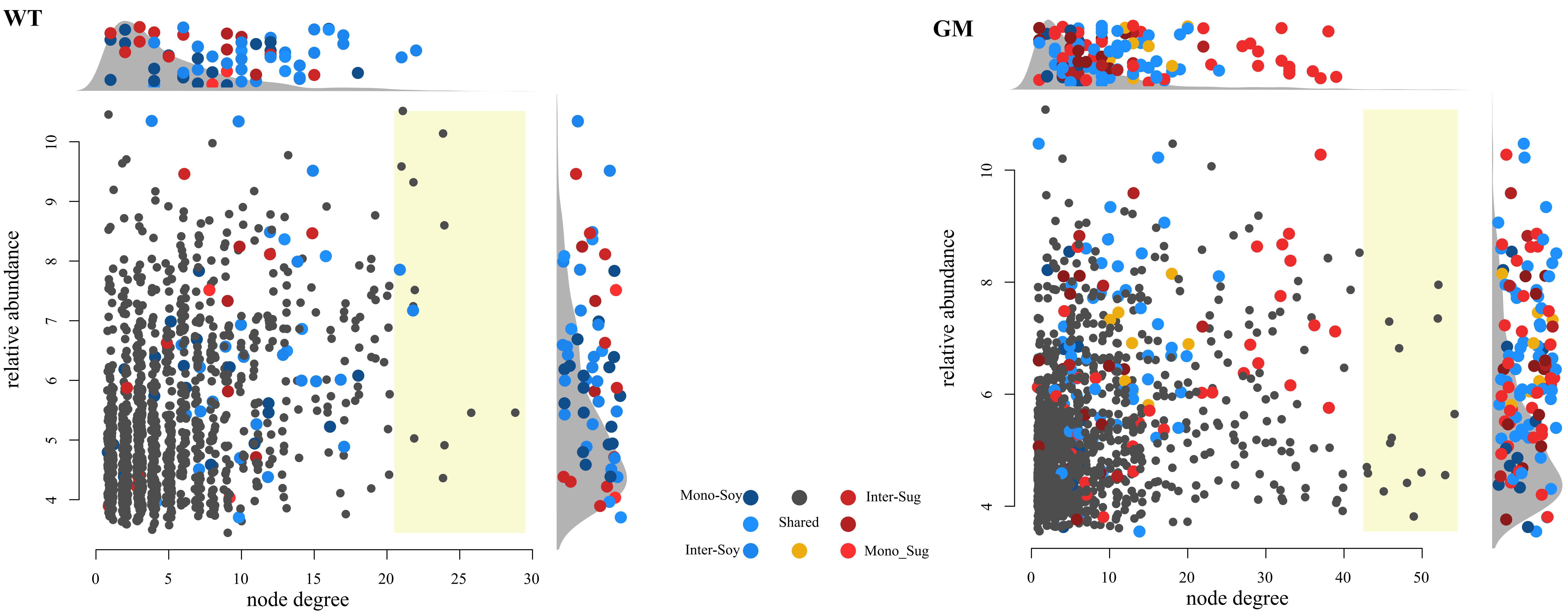

Figure S1: Degree of co-occurrence and abundance of csOTUs. Circles refer to bacteria. OTUs were colored by their association to the different cropping systems. Side panels recapitulate the distributions of co-occurrence degrees and abundance for the csOTUs compared to the density of all.
