# Supplementary figures and images for: Genetically Modified Sugarcane Intercropping Soybean Impact on Rhizosphere Bacterial Communities and Co-occurrence Patterns
Source: Front Microbiol. 2021 Dec 9;12:742341. doi: 10.3389/fmicb.2021.742341 (PMC8713472; doi:10.3389/fmicb.2021.742341)

Figure S2 Response OTUs in each treatment at genus level (Top three)

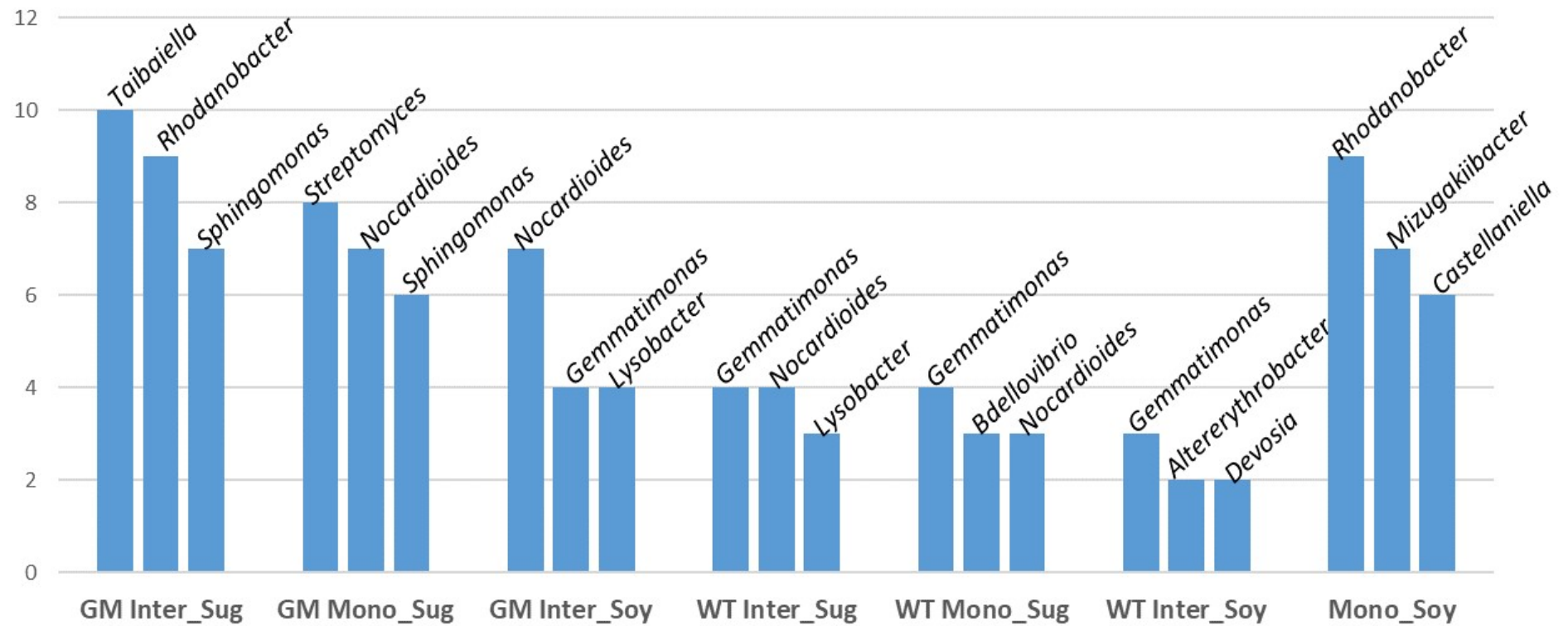

Supplement: Supplementary file 6 [file Data_Sheet_5.PDF]
